# Predictors of Donor‐Site Wound Complications Following Fibula Free Flap Reconstruction

**DOI:** 10.1002/oto2.70126

**Published:** 2025-05-05

**Authors:** Soroush Ershadifar, Angela Colback, Ugur Nur Basmaci, Machelle Wilson, Andrew C. Birkeland, Dustin A. Silverman

**Affiliations:** ^1^ Department of Otolaryngology–Head and Neck Surgery University of California–Davis Sacramento California USA; ^2^ Department of Otorhinolaryngology–Head and Neck Surgery McGovern Medical School, The University of Texas Health Science Center at Houston Houston Texas USA; ^3^ Department of Biostatistics, Epidemiology, and Research Design, Clinical and Translational Science Center University of California–Davis Sacramento California USA; ^4^ Department of Otolaryngology–Head and Neck Surgery University of Cincinnati Cincinnati Ohio USA

**Keywords:** complication, donor‐site, fibula free flap, reconstruction, split thickness skin graft, wound closure

## Abstract

**Objective:**

The fibula free flap (FFF) remains the workhorse flap for head and neck defects necessitating osteocutaneous reconstruction. Although lower extremity angiography, ultrasound (US), and other vascular studies are routinely used for fibula assessment and patient selection, predictors of donor‐site morbidity following harvest remain poorly understood. We sought to investigate the factors associated with FFF donor‐site complications.

**Study Design:**

Retrospective analysis of patients at a tertiary care center.

**Setting:**

Tertiary care center.

**Methods:**

In total, 119 patients undergoing FFF reconstruction during the years 2012 to 2022 were included. Multivariable logistic regression was used to identify independent predictors of soft‐tissue donor‐site wound complications.

**Results:**

A total of 48 (40.3%) patients developed a donor‐site wound complication with an average time to diagnosis of 24 days (±16) following surgery. In multivariable regression, history of alcohol use disorder (*P* = .0083) and method of donor‐site closure (*P* = .0368) were independent predictors of donor‐site wound complications. Split‐thickness skin graft closure was associated with a 146% increased odds of wound complications (odds ratio [OR] = 2.46, 1.11‐5.43, 95% confidence interval). Patient age, body mass index, Charlson comorbidity index, skin paddle size, and Doppler US characteristics were not predictive of postoperative donor‐site morbidity.

**Conclusion:**

Predictors of FFF donor‐site wound complications included history of alcohol abuse and method of donor‐site closure. This study highlights unique lower extremity Doppler US findings in patients undergoing FFF reconstruction in addition to modifiable risk factors associated with fibula donor‐site morbidity and soft‐tissue complications. Our findings underscore the need to critically evaluate wound closure techniques in this population.

Extensive maxillary and mandibular defects are often caused by traumatic injuries or locoregionally advanced head and neck tumors requiring extirpation and reconstruction with composite osteocutaneous free flaps. The goal of mandibular and maxillary reconstruction following complex ablative surgery is to reestablish the form and function of the mid and lower face. Thus, reestablishing premorbid dental occlusion, mastication, communication, airway patency, and optimizing cosmetic outcomes is paramount.^1^ To this end, the fibula free flap (FFF) has been established as the workhorse flap in defects necessitating bone and soft‐tissue components, given its robust bone stock, long vascular pedicle, reliable anatomy, and available skin paddle.^2^

Despite its advantages, donor‐site wound complications are a primary source of morbidity following flap harvest (Figure 1). Complications, including skin graft loss, abscess formation, and ischemia, remain of unique concern with rates approximating 30% in some series.^3^ Although several risk factors, such as diabetes, peripheral vascular disease, and the presence of vascular defects, have previously been described, a paucity of literature examining additional factors that influence FFF donor‐site complications exists.^3^, ^4^, ^5^, ^6^, ^7^, ^8^, ^9^, ^10^ Such complications can lead to impaired function and quality of life for patients, resulting in pain and ambulation restrictions.^11^, ^12^, ^13^

**Figure 1:**
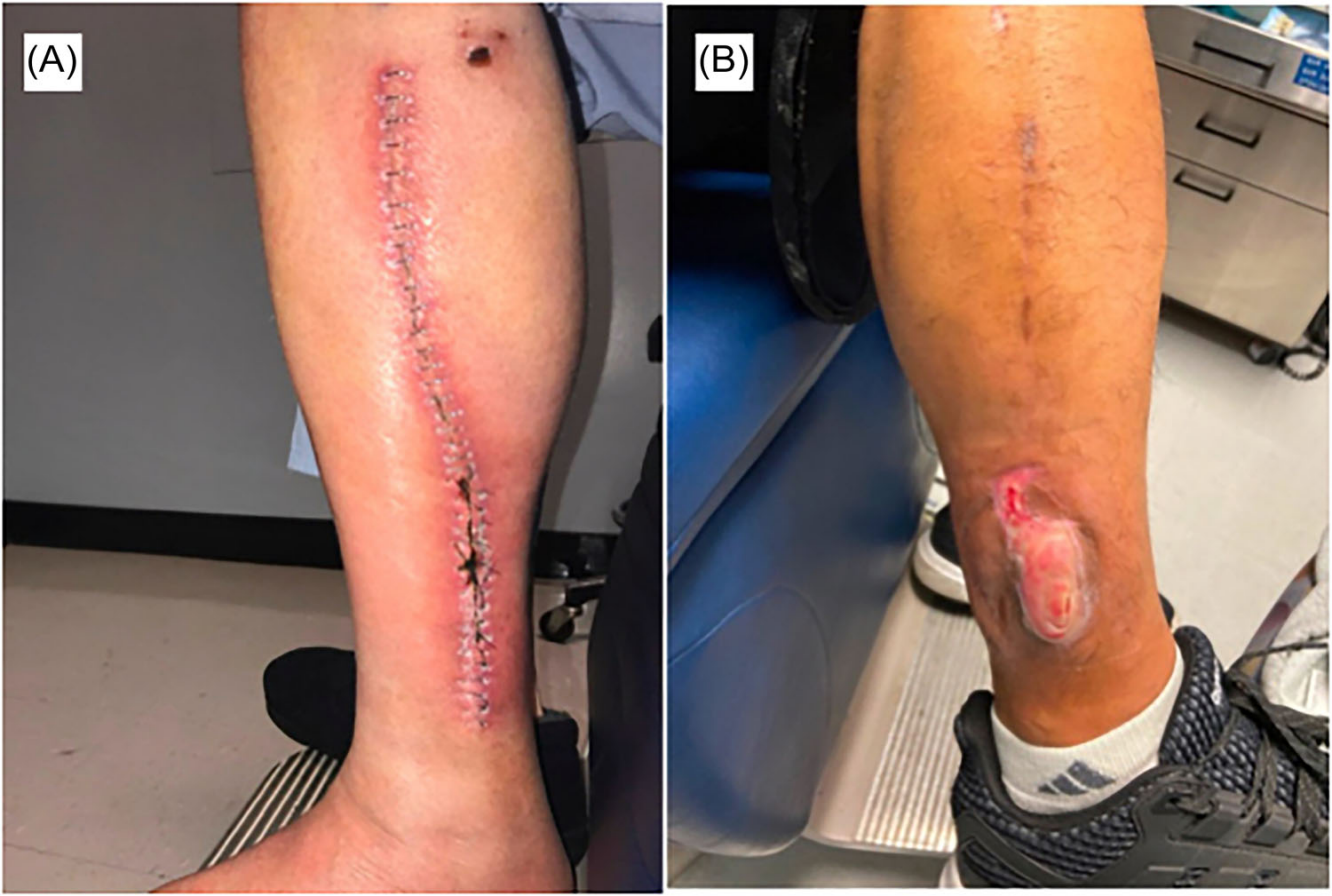
(A) Donor‐site cellulitis in a patient with primary wound closure. (B) Skin dehiscence and partial graft loss in a patient with split‐thickness skin graft closure.

The assessment of lower limb perfusion and vascular health is critically important as the peroneal artery is included during FFF harvest.^14^ Although lower limb perfusion directly affects flap survival as a donor graft, viability of the lower extremity and donor‐site healing remains dependent on the adequacy of remaining posterior and anterior tibial arteries for circulation. Subsequently, the presence of vascular plaques, stenosis, or congenital defects may lead to significant donor‐site morbidity, including wound dehiscence, graft loss, or limb ischemia.^15^

To assess the adequacy of the FFF as a reconstructive option, several measures, including preoperative computed tomography angiography, magnetic resonance angiography, and Doppler ultrasonography (US), have been utilized.^16^, ^17^ At our tertiary care center, lower extremity Doppler US with ankle‐brachial index (ABI) values is the preferred method to objectively analyze lower limb perfusion before flap selection. The ABI value sheds light on perfusion and is a generalized predictor of atherosclerosis in peripheral arteries. Although ABI values ≤ 0.90 have been associated with lower extremity peripheral artery disease (PAD) and atherosclerosis,^17^ abnormally elevated ABI values >1.40 have also been associated with vascular calcification and cardiovascular disease.^18^, ^19^ Futran et al first reported a correlation between ABIs less than 1.0 and arterial occlusive disease, which could jeopardize the flap donor extremity, and suggested the use of an alternative donor site if the ABI value is less than 1.0.^20^

Despite the utility of lower extremity Doppler US to determine FFF candidacy, additional predictors of FFF donor‐site complications remain poorly understood. The primary objective of this study was to identify factors associated with FFF donor‐site wound and soft‐tissue complications. Additionally, we sought to characterize lower extremity Doppler US findings that have not been previously described in the literature.

## Methods

This study is a retrospective cohort study conducted within the Department of Otolaryngology–Head and Neck Surgery at the University of California, Davis, with the approval of the Institutional Review Board before examination of patient charts (IRB # 1529807). Adult patients aged ≥18 years undergoing ablation and FFF reconstruction of both benign and malignant head and neck pathologies were included over the last 10 years (2012‐2022). Patients who underwent an operation without preoperative angiographic evaluation, those without a reported ABI value, and those with less than 12 months of documented clinical follow‐up were excluded from analysis. Charlson comorbidity index (CCI), which classifies the comorbid conditions including diabetes, history of myocardial infarction, congestive heart failure, peripheral vascular disease, cerebrovascular disease, dementia, chronic obstructive pulmonary disease, liver disease, and malignancy (excluding head and neck), was calculated for the patients based on chart review.^21^

In all cases, the FFF was harvested, and the postoperative care pathway was initiated according to the following protocol: according to surgeon preference, an esmarch bandage was applied to the lower extremity, and a tourniquet was inflated to 350 mm Hg. A FFF was then harvested in the standard fashion. Following the harvest, the donor site was closed primarily if able to be completed under minimal tension after placement of two closed‐suction drains into the wound. In cases where primary closure of the donor site was not possible, a split‐thickness skin graft (STSG) was harvested and inset into the wound. An ipsilateral STSG was harvested using a dermatome set at a thickness measuring 0.02 in and inset circumferentially with chromic suture in a running fashion. After incisions were placed into the STSG to allow for fluid egress, a sponge bolster wrapped in xeroform was then secured overlying the STSG. The patient was then placed in a lower extremity immobilization boot; patient ambulation was encouraged starting postoperative day 1, and the boot was able to be removed when lying in the hospital bed. The donor‐site xeroform bolster was removed on postoperative day 6, and photo documentation was obtained. Local wound care was then commenced and included twice‐daily application of a petroleum ointment with a nonadherent dressing secured with a leg wrap below the knee; this was continued until at least the second postoperative week. When feasible, a propeller flap was instead harvested in a manner previously described.^22^, ^23^, ^24^ The decision to harvest a propeller flap was based on whether an appropriate proximal perforator was able to be readily identified, typically as a musculocutaneous perforator through the soleus muscle. Notably, the proximal perforator was able to be utilized in cases when originating from a source other than the donor peroneal vascular pedicle (eg, posterior tibial artery) or, more rarely, when the peroneal vessel was clipped distal to this takeoff without compromising FFF pedicle length.^24^ A donor‐site wound complication was defined as dehiscence, cellulitis, abscess, skin graft loss, tendon exposure, hematoma, seroma, or other complication related to the donor‐site based on the physical examination and as noted in the electronic medical record within the first 60 days postoperatively.

### Statistical Analysis

Summary statistics are presented as mean and standard deviation or percent, as appropriate. Univariate comparisons between treatments were conducted using chi‐square tests, except for age, which was tested using the *t* test. A multivariable logistic regression was fit that included those covariates thought to be clinically important or that were significant in the univariate models. All statistical analyses were conducted using SAS® software for Windows® version 9.4 (SAS Institute Inc.).

## Results

### Cohort Characteristics

In total, 119 patients were included in the study. Baseline cohort characteristics for the patients is identified in Table 1. Sixty‐seven patients (56.3%) were male, and 52 were female (43.6%). The mean age of patients at the time of the surgery was 62.0 years. Seventy‐four patients (62.2%) had a history of tobacco abuse, whereas 45 (37.8%) were identified to have a history of alcohol use disorder. Forty‐two patients (35.3%) had a history of chemotherapy or radiation before surgery as treatment for prior head and neck malignancy. Seventy‐five patients (63.0%) underwent FFF reconstruction following squamous cell carcinoma (SCC) tumor resection, with osteoradionecrosis (16.8%) and ameloblastoma (5.9%) comprising the remaining surgical indications, with highest number of patients. The average CCI value in the patient population was identified to be 0.48 ± 0.82. Overall, 14 (11.8%) patients were identified to have diabetes, and only 4 (3.4%) had a history of peripheral vascular disease. The average ABI value identified on Doppler US before surgery was 1.067 ± 0.096. Doppler studies identified an average of 3.7 ± 1.3 perforators preoperatively in the harvested leg.

**Table 1:** Cohort Characteristics of Patients Undergoing Fibula Free Flap Reconstruction

Patient variable	Number of patients (%)
Mean age at time of surgery (SD)	62 (±13)
Sex	
Male	67 (56.3%)
Female	52 (43.7%)
Race	
Caucasian	95 (79.8%)
African American	3 (2.5%)
Asian/Pacific Islander	6 (5.0%)
Other	15 (12.6%)
History of tobacco abuse	
Yes	74 (62.2%)
No	45 (37.8%)
History of alcohol use disorder	
Yes	45 (37.8%)
No	74 (62.2%)
Charlson comorbidity index (CCI)	
0	80 (67.2%)
1	25 (21.0%)
**≥**2	14 (11.8%)
History of chemotherapy or radiation before FFF	
Yes	42 (35.3%)
No	77 (64.7%)
Location of primary tumor	
Oral cavity	105 (88.2%)
Maxilla	8 (6.7%)
Other (salivary gland, sinonasal, and thyroid)	6 (5.0%)
Pathologic diagnosis	
SCC	75 (63.0%)
Osteoradionecrosis	20 (16.8%)
Ameloblastoma	7 (5.9%)
Trauma	5 (4.2%)
ACC	3 (2.5%)
Sarcoma	2 (1.7%)
Arteriovenous malformation	2 (1.7%)
Adenocarcinoma	1 (0.8%)
Other	4 (3.4%)

Abbreviations: ACC, adenoid cystic carcinoma; FFF, fibula free flap; SCC, squamous cell carcinoma; SD, standard deviation.

The majority of the patients' donor sites were either closed primarily (59.7%) or using STSG closure (37.8%); only three patients underwent propeller flap closure. Average skin paddle size (SPS) among those with osteocutaneous FFF was measured to be 73.75 cm^2^. Among patients who underwent primary donor‐site closure, average SPS size measured 60.78 cm^2^ compared to STSG and propeller flap closure with average of 95.98 and 49.33 cm^2^, respectively. Forty‐eight (40.3%) patients presented with a donor‐site wound complication following FFF harvest, with an average time of diagnosis 24.3 days postoperatively. The average time to ambulation was 2.7 days postoperatively.

### Risk Factors and Post‐FFF Wound Complications

A total of 48 (40.3%) patients developed a donor‐site wound complication with an average time to diagnosis of 24 days (±16) following surgery. Table 2 highlights the donor‐site wound complications within the study cohort. Cellulitis was the most common soft‐tissue complication in 26 patients, followed by wound dehiscence and partial skin graft loss. Table 3 details the characteristics of the 48 patients who developed a post‐FFF donor‐site wound complication. Overall, the history of alcohol use disorder (*P* = .0083) and the method of donor‐site closure (*P* = .0368) presented with statistically significant differences among patients with and without donor‐site complications. These findings persisted in a multivariate model (Table 4). Of these, 33 (68.8%) patients with a donor‐site complication had a prior history of tobacco abuse, whereas 25 (52.1%) had a prior history of alcohol use disorder. In comparison, of the 71 patients who did not develop a donor‐site complication, 41 (57.8%) had a prior history of tobacco abuse, whereas 20 (28.2%) patients had a history of alcohol use disorder.

**Table 2:** Overview of Donor‐Site Wound Complications

Complication	Number of patients (%)^a^
Cellulitis	26 (54.2%)
Wound dehiscence	13 (27.1%)
Partial skin graft loss	13 (27.1%)
Tendon exposure	6 (12.5%)
Abscess	3 (6.3%)
Hematoma	1 (2.1%)
Seroma	1 (2.1%)

^a^ Out of 48 patients who developed a postoperative wound complication, some patients experienced multiple complications.

**Table 3:** Comparisons of Patients by Fibula Donor‐Site Complication

	Number of patients (%)		
Patient variable	Present DSWC	No DSWC	*P*
Mean age at time of surgery (SD), y	64 (±9)	61 (±15)	.1783^a^
Sex			
Male	31 (64.6%)	36 (50.7%)	.1343
Female	17 (35.4%)	35 (49.3%)	
Race			
Caucasian	39 (81.3%)	56 (78.9%)	
African American	1 (2.1%)	2 (2.8%)	
Native American	1 (2.1%)	0	.3247
Asian/Pacific Islander	2 (4.2%)	4 (5.6%)	
Other	3 (6.3%)	9 (12.7%)	
Unknown	2 (4.2%)	0	
History of tobacco abuse			
Yes	33 (68.8%)	41 (57.8%)	.2246
No	15 (31.2%)	30 (42.2%)	
History of alcohol use disorder			
Yes	25 (52.1%)	20 (28.2%)	.0083
No	23 (47.9%)	51 (71.8%)	
Diabetes			
Yes	5 (10.4%)	9 (12.7%)	.7075
No	43 (89.6%)	62 (87.3%)	
History of radiation or chemotherapy before FFF			
Yes	18 (37.5%)	24 (33.8%)	.6788
No	30 (62.5%)	47 (66.2%)	
Doppler study			
Average ABI (harvested leg) [SD]	1.074 (±0.089)	1.063 (±0.10)	.5159^a^
Flap characteristic			
Number of perforators^b^ (SD)	3.73 (1.4548)	3.69 (1.1413)	.8763^a^
Skin paddle harvest size (SD), cm^2^	75.74 (43.84)	72.41 (50.78)	.7101^a^
Method of donor‐site closure			
Primary closure/bilateral advancement flap	22 (45.8%)	49 (69.0%)	
STSG with bolster or wound vacuum	24 (50.0%)	21 (29.6%)	.0368
Propeller flap	2 (4.2%)	1 (1.4%)	

Abbreviations: ABI, ankle‐brachial index; DSWC, donor‐site wound complication; FFF, fibula free flap; SD, standard deviation; STSG, split‐thickness skin graft.

^a^ Independent *t* test.

^b^ Number of perforators on the harvested leg.

**Table 4:** Multivariate Analysis of Factors Associated With Postoperative Donor‐Site Wound Complications

Patient variable	Adjusted odds ratio (95% CI)	*P*
Method of donor‐site closure		
Primary closure/bilateral advancement flap	[Reference]	<.05
STSG	2.449 (1.105‐5.427)	
Propeller flap	3.482 (0.279‐43.438)	
History of alcohol use disorder		
No	[Reference]	<.05
Yes	2.579 (1.105‐5.993)	
History of tobacco abuse		
No	[Reference]	NS
Yes	1.054 (0.445‐2.499)	
Diabetes		
No	[Reference]	NS
Yes	0.8010 (0.251‐2.556)	

Abbreviations: CI, confidence interval; NS, not significant; STSG, split‐thickness skin graft.

## Discussion

Donor‐site wound complications remain a primary source of morbidity following FFF harvest and reconstruction. In this retrospective study, we identified a history of alcohol use disorder and STSG closure to be associated with increased odds of developing a donor‐site soft‐tissue complication following FFF harvest. Alongside these findings, we were able to characterize novel preoperative lower extremity Doppler US findings—ABI and number of perforators—among this patient cohort.

STSG was found to be associated with an increased odds of developing a donor‐site complication in those undergoing FFF. There have been few prior studies evaluating donor‐site complications in FFF patients, which vary in the number of enrolled subjects. In a similar single‐institution study examining 157 patients, Momoh et al failed to identify an association between the method of donor‐site closure with the development of a wound complication.^25^ However, there were notable differences compared to the current study, including a shorter study timeline (30 days), lack of routine angiography amongst all patients, and a higher proportion of those undergoing STSG placement, which may explain the differences in outcomes. In a meta‐analysis including five studies on 185 FFFs, the authors found the weighted mean rate of complications in those undergoing an STSG to be almost two times higher compared to primary wound closure.^26^ Higher complication rates associated with this method may be inherent to the limitations of skin grafting, secondary to poor adherence to bone or tendinous tissue, and high susceptibility to trauma, likely secondary to ambulation in this population. Interestingly, the time to ambulation amongst our cohort was 2.7 days, whereas this averaged greater than 5 days in the Momoh et al cohort; neither of these series found an association between time to ambulation and development of donor‐site wound complications. McCrary et al demonstrated that a quality improvement project focused on early ambulation among FFF patients without postoperative boot placement following FFF was associated with reduced length of stay, increased mobility independence, and improved discharge to home rates, though the presence of wound complications was not assessed in their cohort.^27^ Although institutional practices on wound care differ, future multi‐institutional studies are needed to better evaluate these relationships. Selecting the ideal method of donor‐site closure is multifactorial and dependent on surgeons' preferences and intraoperative assessment, for example, whether or not a lower extremity FFF site may be closed primarily with minimal tension so as to avoid dehiscence or compartment syndrome. In making such decisions, the width of the defect, age of the patient, and laxity of skin are all important factors to be considered. Although some authors have suggested that defects with a width smaller than 6 cm should be closed primarily,^28^ others have suggested more conservative widths, such as only smaller than 4 cm, due to fears of compartment syndrome.^29^, ^30^ To address soft‐tissue complication rates associated with STSG coverage of the FFF donor site, surgeons may consider using a full‐thickness skin graft (FTSG), locoregional flap, or harvesting a thicker STSG, particularly in those at higher risk of delayed wound healing. When harvesting a STSG for FFF donor‐site coverage, it is important to ensure appropriate graft size for appropriate soft‐tissue coverage. Additionally, there is emerging evidence for the use of biologic healing agents such as an acellular dermal matrix or dermal substitute to enhance recovery, though further study is required to elucidate their role in donor‐site recovery during head and neck reconstructive surgery.^31^

The current study was also able to identify an association between a history of alcohol use disorder with the development of donor‐site complications following FFF. The impact of chronic and acute alcohol exposure on wound healing has been well‐documented. Genther and Gourin found a similar association in those undergoing head and neck cancer surgery.^32^ Other series have also found similar results in concordance with the present cohort regarding the relationship of alcohol use and the development of postsurgical wound complications, not limited to head and neck cancer regions.^33^, ^34^, ^35^, ^36^ Chronic alcohol exposure may result in decreased deposition of protein and collagen, impairing wound healing and higher susceptibility to postoperative infection. Given the high prevalence of alcohol use disorder in patients with head and neck cancer, it is important to grant special attention to those at higher risk during the perioperative period. Moreover, adherence to a perioperative alcohol abstinence contract has been shown to significantly affect healing outcomes, with higher rates of alcohol withdrawal, delirium, cellulitis, and wound dehiscence among those in a noncontracted compared to a contracted group.^37^ We did not find an association between other comorbidities, including diabetes or history of tobacco abuse, with the development of lower extremity wound complications. This is likely secondary due to the strong impact of these conditions with atherosclerosis and peripheral vascular disease, which would have likely excluded a patient from undergoing FFF.^6^, ^38^ Notably, some prior studies have also failed to identify an association between these factors, likely due to this underlying mechanism.^25^

Our study is one of the first to describe unique lower extremity Doppler US findings for patients undergoing FFF harvest. With respect to donor‐site wound complications, we did not identify an association between ABI values and the presence of complications in our cohort. These findings are limited by the exclusion of abnormal ABI values during the surgical candidacy process. Alongside ABI, we did not find an association between the number of available perforators and wound complications—with the number of perforators ranging from 1 to 7. Interestingly, our cohort included a number of patients with ABI values less than 1.0, which has been traditionally used as a cutoff for determining FFF candidacy.^20^ Although outside the scope of this study, the decision to undergo FFF in these patients was made based on specific patient and oncologic factors such as length of the bone required for reconstruction, and further studies are needed to better evaluate the impact of ABI and other comorbidities on flap outcomes.

Ultimately, we identified STSG and a history of alcohol use disorder as factors associated with an increased risk of donor‐site wound complications in patients undergoing FFF. Our findings can assist surgeons in identifying patients at higher risk for developing donor morbidity after surgery, allowing for potential adjustments in postoperative care, such as extended wound management and closer observation.

Several limitations are recognized in this study. First, our study is limited in its design as a retrospective chart review. The timing of postoperative ambulation varied slightly between patients and may also affect the presence of donor‐site morbidity. Although the presence of several comorbidities, such as diabetes and PAD, are relative contraindications to FFF harvest, our study was not specifically designed to assess the possible impact of sarcopenia on wound healing outcomes. Prior series have focused on important long‐term functional donor‐site morbidity, including leg weakness, ankle instability, range of motion, and toe contracture outcomes with favorable results; however, objective physical therapy metrics were not routinely available among our cohort and thus considered outside the scope of our study.^13^, ^25^, ^26^ Although harvested STSG thickness was standard across our cohort, future studies may examine the impact of various thickness levels on ultimate graft take. Finally, our study is limited in its power and number of participants. Future studies should expand the number of patients through multidisciplinary collaboration to limit institutional or surgeon‐related factors that may influence the final results.

## Conclusions

Donor‐site complications following FFF harvest are a significant source of morbidity despite appropriate patient and flap selection following preoperative lower extremity Doppler US. History of alcohol use disorder and method of donor‐site closure, specifically STSG use, were predictive of donor‐site wound complications. This study highlights unique lower extremity Doppler US findings in patients undergoing FFF reconstruction in addition to modifiable risk factors associated with fibula flap donor‐site morbidity. These findings underscore the need to critically evaluate wound‐closure techniques in this population to reduce and prevent complications that may result in undesirable outcomes for patients undergoing FFF reconstruction.

## Author Contributions

**Soroush Ershadifar**, idea, design, study execution, analysis, and manuscript preparation; **Angela Colback**, analysis and manuscript preparation; **Ugur Nur Basmaci**, design, study execution, and manuscript preparation; **Machelle Wilson**, analysis and manuscript preparation; **Andrew C. Birkeland**, idea, design, and manuscript preparation; **Dustin A. Silverman**, idea, design, study execution, analysis, and manuscript preparation.

## Disclosures

### Competing interests

The authors of this work have no declarations of interest.

### Funding source

The authors of this work have no financial disclosures.
